# Parity, hormones and breast cancer subtypes - results from a large nested case-control study in a national screening program

**DOI:** 10.1186/s13058-016-0798-x

**Published:** 2017-01-23

**Authors:** Merete Ellingjord-Dale, Linda Vos, Steinar Tretli, Solveig Hofvind, Isabel dos-Santos-Silva, Giske Ursin

**Affiliations:** 1 Cancer Registry of Norway, Oslo, Norway; 2 Department of Non-Communicable Disease Epidemiology, London School of Hygiene and Tropical Medicine, London, UK; 3 University of Oslo, Oslo, Norway; 4 University of Southern California, Los Angeles, CA USA

**Keywords:** Reproductive factors, Hormone therapy, Breast cancer subtypes

## Abstract

**Background:**

Breast cancer comprises several molecular subtypes with different prognoses and possibly different etiology. Reproductive and hormonal factors are associated with breast cancer overall, and with luminal subtypes, but the associations with other subtypes are unclear. We used data from a national screening program to conduct a large nested case-control study.

**Methods:**

We conducted a nested case-control study on participants in the Norwegian Breast Cancer Screening Program in 2006 − 2014. There was information on estrogen receptor (ER), progesterone receptor (PR) and human epidermal growth factor receptor 2 (HER2) for 4748 cases of breast cancer. Breast cancer subtypes were defined as luminal A-like (ER+ PR+ HER2-), luminal B-like (ER+ PR- HER2- or ER+ PR+/PR-HER2+), HER2-positive (ER- PR- HER2+) and triple-negative (ER- PR- HER2-). Conditional logistic regression was used to estimate odds ratios (ORs) of breast cancer associated with age at first birth, number of pregnancies, oral contraceptive use, intrauterine devices and menopausal hormone therapy. Analyses were adjusted for age, body mass index, education, age at menarche, number of pregnancies and menopausal status.

**Results:**

Number of pregnancies was inversely associated with relative risk of luminal-like breast cancers (*p*-trend ≤0.02), and although not statistically significant, with HER2-positive (OR = 0.60, 95% CI 0.31–1.19) and triple-negative cancer (OR = 0.70, 95% CI 0.41–1.21). Women who had ≥4 pregnancies were at >40% lower risk of luminal-like and HER2-positive cancers than women who had never been pregnant. However, there was a larger discrepancy between tumor subtypes with menopausal hormone use. Women who used estrogen and progesterone therapy (EPT) had almost threefold increased risk of luminal A-like cancer (OR = 2.92, 95% CI 2.36–3.62) compared to never-users, but were not at elevated risk of HER2-positive (OR = 0.88, 95% CI 0.33–2.30) or triple-negative (OR = 0.92, 95% CI 0.43 − 1.98) subtypes.

**Conclusions:**

Reproductive factors were to some extent associated with all subtypes; the strongest trends were with luminal-like subtypes. Hormone therapy use was strongly associated with risk of luminal-like breast cancer, and less so with risk of HER2-positive or triple-negative cancer. There are clearly some, but possibly limited, etiologic differences between subtypes, with the greatest contrast between luminal A-like and triple-negative subtypes.

**Trial registration:**

Not applicable.

## Background

There is substantial evidence for a role of female hormones in the etiology of breast cancer. Reproductive factors, such as early age at menarche, nulliparity, and late age at first birth [1–3] are all believed to be associated with breast cancer risk through hormonal mechanisms. Current use of oral contraceptives is associated with some increased risk of breast cancer [4–6]. Likewise, combined postmenopausal hormone therapy (estrogen and progesterone), increases the risk of breast cancer [7–16]. However, breast cancer consists of several molecular subtypes that have very different prognoses [17–23]. It is less clear whether these various subtypes have different etiologies. There have been two main challenges in previous literature: the various definitions used to define breast cancer subtypes, and the lack of power to examine the effect on all subtypes.

In earlier studies, subtypes were defined by immunohistochemical analysis (IHC) of the two hormone receptors only, i.e. the estrogen receptor (ER) and the progesterone receptor (PR) [24–31]. It is only in recent years that studies have been conducted incorporating data on human epidermal growth factor receptor 2 (HER2) status [32–38], with a few having included other additional molecular markers [3, 34, 39–44]. However, most studies so far have not been able to run molecular expression studies on a large scale. This may not be necessary however, because there is a large degree of overlap between the immunohistochemical subtypes defined by ER, PR, and HER2 status and those identified by molecular expression studies [18–21, 45]. However, a challenge has been that investigators have used various definitions and specific markers to define breast cancer subtypes, such as luminal B tumors. Further, using the molecular terms luminal A, B, or basal gives the impression that they are defined using molecular expression markers, whereas they are based on IHC. Investigators at the St. Gallen meeting in 2013 therefore suggested that the term “like” should be added to the molecularly derived “luminal” subtype names (e.g. “luminal A-like”, “luminal B-like”) to indicate that IHC was used to define these subtypes [46] and hence, that they are proxies of the molecular subtypes.

Another challenge has been that many studies have not had adequate power to assess the effect of risk factors on all subtypes, which makes it difficult to determine the effect of risk factors on the less common subtypes. The overall published evidence [7, 8, 10, 47–50] seems to be consistent with luminal A-like cancers having a hormonal etiology, but the association between hormonal factors and other subtypes, in particular luminal B-like disease, HER2-positive disease, and triple-negative cancer, is less clear. Specifically, reproductive factors such as parity and early age at first birth have been associated with reduced risk of luminal A-like disease. There is less evidence of a protective effect of parity on luminal B-like and HER2-positive cancers and parity has consistently been found not to protect against triple-negative disease [7, 47–50]. There is some, albeit inconsistent, evidence that older age at menarche and breastfeeding may protect against all subtypes [7, 8, 47, 49–52] suggesting that these protective effects may work through non-hormonal mechanisms. The use of menopausal hormone therapy has been consistently associated with an increased risk of luminal A-like breast cancer, but the evidence is less clear for risk of luminal B-like, HER2-positive and triple-negative breast cancer [10, 47, 49].

We decided to take advantage of a national screening program in Norway to examine potential associations between reproductive and hormonal factors and the various breast cancer subtypes - specifically, luminal A-like, luminal B-like HER2-negative, luminal B-like HER2-positive, HER2-positive, and triple-negative disease - in a large study. We therefore conducted a large nested case-control study within the Norwegian Breast Cancer Screening Program.

## Methods

### Study population

The Cancer Registry of Norway (CRN) is responsible for the administration of the Norwegian Breast Cancer Screening Program [53]. Women aged 50–69 years are invited to undergo two-view mammography screening every 2 years. From August 2006, women, who underwent mammographic screening in the national program were asked to complete a questionnaire on a number of standard breast cancer risk factors, and another questionnaire on current exposure variables at subsequent screenings. For the current study cohort, women who had participated in the Norwegian Breast Cancer Screening Program during 2006 to 2014 and had completed these questionnaires were eligible for inclusion. The study cohort comprised a total of 344,348 eligible women. Information on cancer cases was obtained through linkage to the population-based CRN records using the unique 11-digit personal identification number assigned to all residents at time of birth. This linkage also included information on vital status, including date and cause of death/date of emigration if applicable. Reporting of cancer to the CRN is mandatory by law, and the registry is considered to be 98.8% complete [54].

All women aged 50–69 years who are included in the National Population Registry are invited to undergo screening as part of the Norwegian Breast Cancer Screening Program every 2 years. The average attendance rate in each round is about 76%.

We conducted a nested case-control study within the study cohort. Only women with no history of ductal carcinoma in situ prior to the study start (1 January 2006) and no history of diagnosis of another invasive cancer (except non-melanoma skin cancer) were eligible for the study. The cases were women diagnosed with a first occurrence of invasive breast cancer (ICD10: C50) during the study period, with information on ER, PR, and HER2 status (see subsequent text). For each woman with breast cancer, we randomly selected five controls individually matched to cases by year of birth (+/- 3 years) and year of last screening before breast cancer diagnosis (+/- 3 years). Controls had to be alive and resident in the country at the time of the diagnosis of breast cancer in the matching case. We ended up with 6471 patients with breast cancer (cases) and 32,355 controls. The study was approved by the Regional Committee for Medical and Health Research Ethics in the South-East Health Region of Norway.

### Tumor receptor status ascertainment

Information on ER, PR and HER2 status was assessed by IHC and extracted from pathology reports submitted to the CRN. From 2006 to January 2012, tumors were classified as ER-negative if there was <10% reactivity. From February 2012 onwards, the threshold for ER-negative tumors was changed to <1% reactivity as a result of change in the treatment protocols for patients attending clinics in Norway. We used these official thresholds. PR-negative tumors were defined as those with reactivity of <10%, and PR-positive tumors as those with reactivity ≥10%. HER2 expression status was determined at each laboratory with IHC and/or in situ hybridization. Cases with no (0) or weak (1+) immunostaining were defined as HER2-negative, while cases with moderate (2+) or strong immunostaining (3+) were defined as HER2-positive. In situ hybridization (fluorescence (FISH), chromogenic (CISH), or silver (SISH) in situ hybridization methods) was usually used to confirm HER2 status if IHC yielded 2+ results. If IHC was 2+ and FISH, CISH, or SISH were missing, or if IHC was missing but FISH, CISH, or SISH were positive, the tumor was classified as HER2-positive. If IHC was 2+ and FISH, CISH, and SISH were negative, the tumor was regarded as HER2-negative. Data on Ki-67 were recorded by the CRN from late 2011 and therefore, we did not include this marker in our analysis.

### Risk factors

Our primary exposures of interest were hormonal risk factors including reproductive factors (age at first birth, number of pregnancies, breastfeeding, menopausal status), and other hormonal factors (use of oral contraceptives, intrauterine devices (IUD) and menopausal hormone therapy use). Menopausal hormone therapy use included use of estrogen alone (estrogen therapy, ET), or use of combined estrogen and progestin therapy (EPT). For current exposures we chose the questionnaire at the last screening before breast cancer diagnosis for the cases and the corresponding time for the controls. If the questionnaire or any values were missing, information was used from the previous screening questionnaire. Menopausal status was defined according to whether or not women were still menstruating, or whether they menstruated irregularly. Menopausal age was defined as the age when menstruation ended.

### Confounders and missing values

We considered age at screening (50–54, 55–59, 60–64, or 65–70 years), body mass index (BMI) at screening (≤22, 23–25, 26-28, or >28), education (no education/primary school, high school, or bachelor’s/master’s degree), age at menarche (9–12, 13, 14, or 15–18 years), number of pregnancies lasting at least six months (never pregnant, 1, 2, 3, or ≥4) and menopausal status (premenopausal, perimenopausal, or postmenopausal) as potential confounders and adjusted for these when appropriate. We tried to control more tightly for age at screening using a continuous variable, but the results remained largely the same, and we therefore retained the 5-year categories.

Women with missing values for an exposure variable were excluded from the analyses of that variable, while women with missing information on the potential confounding variables listed previously were excluded from all analyses. Of the 6471 patients with breast cancer, we excluded women due to missing information on the following variables: BMI (*n* = 532 patients), age at menarche (*n* = 298 patients), education (*n* = 66 patients), number of pregnancies (*n* = 164 patients), and menopausal status (*n* = 59 patients). This left us with 5352 women with breast cancer (cases) for analysis. Of the 32,355 controls, we excluded controls based on missing information on the following: BMI (*n* = 3296 controls), age at menarche (*n* = 1641 controls), education (*n* = 371 controls), number of pregnancies (*n* = 779 controls), and menopausal status (*n* = 336 controls). This left us with 25,932 controls for analysis.

The cases were categorized by breast cancer subtype using a modified version of the classification of clinically defined subtypes proposed at the St. Gallen meeting in 2013 [46]. Of the 5352 cases of breast cancer, 604 had unknown hormone receptor (i.e. ER and/or PR) and HER2 status or could not be classified into the breast cancer subtypes. There were 4748 women with breast cancer classified into the following subtypes: 2985 women with luminal A-like breast cancer (ER+ PR+ HER2-), 758 women with luminal B-like HER2-negative breast cancer (ER+ PR- HER2-), 396 women with luminal B-like HER2-positive breast cancer (ER+ PR+/PR- HER2+), 223 women with HER2-positive breast cancer (ER- PR- HER2+) and 386 women with triple-negative breast cancer (ER- PR- HER2-). As we did not have Ki-67 results, we conducted sensitivity analysis where we added grade to the luminal subtype definitions in an attempt to better separate out these subtypes, using the definitions from the St. Gallen 2013 meeting [46]. In this analysis luminal A-like subtype was defined as ER+ PR+ HER2-, low or medium grade, luminal B-like HER2-negative as ER+ PR- HER2-, high grade, and luminal B-like HER2-positive as ER+ PR-/PR+ HER2+, any grade.

### Statistical analyses

We used conditional logistic regression to estimate odds ratios (with 95% confidence intervals (CI)) as a measure of relative risk associated with various exposures. Trend tests were performed by fitting ordinal values corresponding to exposure categories and testing whether the slope coefficient differed from zero. To test for heterogeneity between breast cancer subtypes we ran case–case analyses, comparing each subtype to the luminal-A-like subtype. We used likelihood ratio tests comparing the likelihood ratio of the case–case model with confounders only to that of the adjusted case–case model with the exposure variable. We considered a two-sided *p* value <0.05 as statistically significant.

Because we used conditional logistic regression we included all women in the risk estimates (e.g. women who were never pregnant were included in analysis of age at first birth, women who never used oral contraceptives (OC) were included in analysis of age at the start of OC, and premenopausal women were included in analysis of age at menopause), but they were not included in the trend test (dummy variables were added into the analysis).

## Results

BMI, age at first birth, education, age at menopause, duration of use of oral contraceptives and intrauterine devices, and menopausal hormone therapy use were positively associated with overall breast cancer risk (i.e. all subtypes combined) whereas age at menarche, number of pregnancies and postmenopausal status were associated with a decreased risk (Table 1).

**Table 1:** Adjusted odds ratios (ORs) and 95% confidence intervals (CI) for association between breast cancer overall and education and reproductive and hormonal risk factors

Characteristics	Overall				
	Total (*n*)	Controls (*n*)	Cases (n)	OR*	95% CI
Education					
No education/primary school	8584	7192	1392	1	Ref
High school	12,781	10,582	2199	1.07	(0.99, 1.15)
University Bachelor’s degree	6179	5081	1098	1.11	(1.01, 1.22)
University Master’s degree	3740	3077	663	1.10	(0.98, 1.22)
*p*-trend*				0.03	
BMI (kg/m^2^)					
< = 22	5141	4333	808	1	Reference
23–25	9655	8075	1580	1.07	(0.98, 1.18)
26–28	8131	6694	1437	1.19	(1.08, 1.32)
>28	8357	6830	1527	1.23	(1.12, 1.36)
*p*-trend				<0.0001	
Age at menarche (years)					
9–12	9296	7615	1681	1	Reference
13	8628	7157	1471	0.93	(0.86, 1.01)
14	7704	6439	1265	0.89	(0.82, 0.97)
15–18	5656	4721	935	0.90	(0.82, 0.98)
*p*-trend				0.01	
Age at first birth (years)					
13–20	8413	7085	1328	1	Reference
21–22	4994	4170	824	1.04	(0.94, 1.15)
23–25	6673	5580	1093	1.02	(0.93, 1.12)
26–30	5440	4497	943	1.09	(0.98, 1.20)
31–50	2070	1641	429	1.29	(1.12, 1.48)
Never given birth (nulliparous)	2730	2144	586	1.73	(1.49, 2.01)
*p*-trend^a^				0.003	
Number of pregnancies lasting 6+ months					
0	2730	2144	586	1	Reference
1	3584	2911	673	0.83	(0.73, 0.94)
2	13499	11000	2301	0.76	(0.68, 0.84)
3	8307	6956	1351	0.71	(0.63, 0.79)
>4	3164	2723	441	0.59	(0.51, 0.68)
*p*-trend				<0.0001	
Parous women only					
Duration of breastfeeding (months)					
Parous no breastfeeding	2201	1840	361	1	Reference
1–6	6872	5775	1097	0.97	(0.84, 1.11)
7–12	6662	5452	1210	1.16	(1.01, 1.34)
13–20	5466	4544	922	1.08	(0.93, 1.25)
21–30	3620	3043	577	1.04	(0.88, 1.22)
>30	2067	1736	331	1.10	(0.91, 1.33)
*p*-trend				0.14	
Menopausal status					
Premenopausal	2568	2035	533	1	Reference
Perimenopausal	2279	1876	403	0.73	(0.63, 0.86)
Postmenopausal	26437	22000	4416	0.66	(0.59, 0.75)
*p*-trend				<0.0001	
Age at menopause (years)					
<47	5412	4595	817	1	Reference
47–49	5151	4324	827	1.10	(0.99, 1.22)
50–52	9831	8218	1613	1.13	(1.03, 1.24)
>52	6663	5508	1155	1.15	(1.04, 1.28)
Premenopausal	2568	2035	533	1.81	(1.57, 2.10)
*p*-trend^b^				0.01	
Age at start of oral contraceptives (years)					
14–18	3097	2577	520	1	Reference
19–20	3356	2686	670	1.19	(1.02, 1.38)
21–24	4190	3490	700	0.95	(0.82, 1.11)
25–50	4307	3590	717	0.91	(0.77, 1.07)
Never used	14532	12000	2443	0.95	(0.85, 1.07)
*p*-trend^a^				0.06	
Duration of oral contraceptives (years)					
Never used	14532	12000	2443	1	Reference
<2	4019	3405	614	0.89	(0.81, 0.99)
2–5	3758	3120	638	1.02	(0.92, 1.13)
6–10	3453	2834	619	1.10	(0.99, 1.22)
>10	2718	2203	515	1.11	(1.00, 1.25)
*p*-trend				0.01	
Age at start of intrauterine device (years)					
14–28	1340	1120	220	1	Reference
29–35	1422	1174	248	1.03	(0.84, 1.27)
36–42	1193	965	228	1.18	(0.95, 1.46)
43–50	1052	859	193	1.06	(0.85, 1.33)
Never used	21734	18000	3693	0.99	(0.84, 1.16)
*p*-trend^a^				0.27	
Duration of intrauterine device (years)					
Never used	21734	18000	3693	1	Reference
<2	729	612	117	0.92	(0.75, 1.14)
2–5	969	791	178	1.07	(0.90, 1.28)
6–10	1403	1158	245	1.10	(0.95, 1.28)
>10	1753	1432	321	1.14	(0.99, 1.30)
*p*-trend				0.03	
Hormone therapy use					
Never	15688	13000	2487	1	Reference
Past	10548	8656	1892	1.16	(1.08, 1.25)
Estrogen current	1468	1219	249	1.03	(0.88, 1.20)
Estrogen and progesterone current	953	661	292	2.32	(1.97, 2.72)
*p*-trend				<0.0001	
Duration of hormone therapy (years)					
Never used	15688	13000	2487	1	Reference
< =3	3636	3066	570	1.00	(0.90, 1.10)
4–8	2449	2013	436	1.17	(1.03, 1.30)
>8	4060	3151	909	1.58	(1.42, 1.70)
*p*-trend				<0.0001	
Duration of estrogen and progesterone therapy (years)					
Never used	15688	13000	2487	1	Reference
< =5	4288	3552	736	1.13	(1.02, 1.25)
>5	4189	3238	951	1.61	(1.46, 1.78)
*p*-trend				<0.0001	

^a^The category *Never used* was not included in the analysis of *p*-trend. bThe category *Premenopausal* was not included in the analysis of *p*-trend. *p for trend and OR mutually adjusted for age (50–54, 55–59, 60–64, 65–70 years at screening), body mass index (BMI) (≤22, 23–25, 26–28, >28 at screening), education (no education/primary school, high school, bachelor’s or master’s degree), age at menarche (9–12, 13, 14, 15–18 years), number of pregnancies (0, 1, 2, 3, ≥4), menopausal status (premenopausal, perimenopausal, postmenopausal)

Number of pregnancies was inversely associated with risk of breast cancer overall and risk of several breast cancer subtypes (Table 2). Compared to women who had never been pregnant, those with ≥4 pregnancies had about 40% or lower risk of developing luminal-like breast cancer, and all three tests for trend were statistically significant (Table 2). There were similar, but non-significant protective associations also with HER2-positive and triple-negative tumors, but neither the point estimate for the top category (≥4 pregnancies: OR = 0.60, 95% CI 0.31–1.19 and OR = 0.70, 95% CI 0.41–1.21, respectively), nor the trend for number of pregnancies was statistically significant. The test for heterogeneity comparing triple-negative cancer to luminal A-like cancer was statistically significant.

**Table 2:** Adjusted odds ratios (OR) and 95% confidence intervals (CI) for association between breast cancer subtypes associated and reproductive factors

	Luminal A-like				Luminal B-like HER2-negative				Luminal B-like HER2-positive				HER2-positive				Triple-negative			
	ER+ PR+ HER2-				ER+ PR- HER2-				ER+ PR+/PR- HER2+				ER- PR- HER2+				ER- PR- HER2-			
	Controls (*n*)	Cases (*n*)	OR*	95% CI	Controls (*n*)	Cases (*n*)	OR	95% CI	Controls (*n*)	Cases (*n*)	OR	95% CI	Controls (*n*)	Cases (*n*)	OR	95% CI	Controls (*n*)	Cases (*n*)	OR	95% CI
Age at first birth (years)																				
13–20	3951	746	1	Reference	1001	169	1	Reference	553	96	1	Reference	279	54	1	Reference	495	95	1	Reference
21–22	2277	460	1.04	(0.91, 1.18)	620	120	1.22	(0.93, 1.59)	305	51	0.97	(0.65, 1.43)	170	32	0.97	(0.58, 1.62)	326	65	1.10	(0.77, 1.57)
23–25	3152	591	0.97	(0.85, 1.10)	759	157	1.29	(1.00, 1.67)	437	92	1.27	(0.90, 1.78)	222	36	0.75	(0.46, 1.22)	393	86	1.27	(0.89, 1.81)
26–30	2473	547	1.12	(0.98, 1.28)	647	141	1.30	(0.99, 1.71)	343	62	1.21	(0.82, 1.78)	216	41	0.97	(0.59, 1.58)	318	63	1.16	(0.79, 1.70)
31–50	937	238	1.19	(0.99, 1.43)	223	60	1.60	(1.10, 2.32)	127	32	1.64	(0.98, 2.74)	63	23	1.67	(0.89, 3.12)	120	29	1.47	(0.88, 2.47)
Never (nulliparous)	1199	328	1.76	(1.44, 2.15)	303	89	1.96	(1.31, 2.91)	164	49	2.74	(1.57, 4.78)	84	27	1.82	(0.85, 3.88)	144	34	1.58	(0.89, 2.81)
*p*-trend^a^			0.07				0.01				0.06				0.49				0.16	
*p*-heterogeneity^#^							0.19				0.19				0.31				0.68	
Number of pregnancies lasting 6+ months																				
0	1199	328	1	Reference	303	89	1	Reference	164	49	1	Reference	84	27	1	Reference	144	34	1	Reference
1	1596	397	0.91	(0.77, 1.08)	426	84	0.68	(0.48, 0.96)	229	42	0.53	(0.33, 0.86)	128	20	0.50	(0.26, 0.96)	224	41	0.70	(0.42, 1.16)
2	6266	1302	0.76	(0.66, 0.88)	1602	323	0.68	(0.52, 0.90)	842	185	0.71	(0.49, 1.04)	426	97	0.78	(0.47, 1.29)	813	158	0.77	(0.50, 1.18)
3	3883	721	0.66	(0.57, 0.77)	950	197	0.69	(0.52, 0.93)	528	89	0.54	(0.36, 0.82)	319	60	0.61	(0.36, 1.03)	477	117	0.98	(0.63, 1.53)
>4	1518	237	0.56	(0.46, 0.68)	383	65	0.58	(0.40, 0.84)	225	31	0.42	(0.25, 0.70)	98	19	0.60	(0.31, 1.19)	196	36	0.70	(0.41, 1.21)
*p*-trend			<0.0001				0.02				0.002				0.19				0.88	
*p*-heterogeneity							0.31				0.42				0.34				0.03	
Parous women only																				
Duration breastfeeding (months)																				
No breastfeeding	997	204	1	Reference	260	51	1	Reference	146	33	1	Reference	78	10	1	Reference	135	24	1	Reference
1–6	3145	607	0.95	(0.79, 1.15)	831	170	1.01	(0.70, 1.45)	449	74	0.66	(0.40, 1.07)	243	37	1.42	(0.63, 3.18)	467	80	1.01	(0.60, 1.71)
7 − 12	3108	663	1.09	(0.91, 1.31)	763	158	1.05	(0.73, 1.51)	427	98	0.83	(0.51, 1.33)	215	52	2.25	(1.02, 4.99)	359	95	1.65	(0.98, 2.78)
13 − 20	2583	496	1.02	(0.83, 1.24)	654	130	0.98	(0.67, 1.44)	343	77	0.90	(0.54, 1.49)	156	48	2.51	(1.11, 5.68)	308	69	1.29	(0.74, 2.23)
21 − 30	1700	319	1.03	(0.83, 1.28)	399	79	0.97	(0.64, 1.46)	240	37	0.60	(0.33, 1.10)	147	28	1.78	(0.74, 4.28)	222	44	1.18	(0.65, 2.15)
>30	966	195	1.15	(0.89, 1.48)	250	48	1.03	(0.63, 1.69)	121	16	0.69	(0.33, 1.47)	70	13	1.82	(0.67, 4.97)	123	24	1.19	(0.58, 2.44)
*p*-trend			0.22				0.90				0.63				0.12				0.52	
*p*-heterogeneity							0.85				0.25				0.56				0.84	
Menopausal status																				
Premenopausal	1171	351	1	Reference	208	47	1	Reference	173	32	1	Reference	100	18	1	Reference	130	29	1	Reference
Perimenopausal	1055	229	0.61	(0.50, 0.74)	225	50	0.95	(0.60, 1.51)	152	26	0.86	(0.47, 1.54)	79	20	1.41	(0.67, 2.94)	143	37	1.13	(0.64, 1.99)
Postmenopausal	12000	2405	0.51	(0.43, 0.60)	3231	661	0.84	(0.58, 1.23)	1663	338	1.11	(0.69, 1.78)	876	185	1.21	(0.66, 2.23)	1581	320	0.82	(0.50, 1.33)
*p*-trend			<0.0001				0.30				0.46				0.71				0.21	
*p*-heterogeneity							<0.0001				0.01				0.001				0.003	
Age at menopause (years)																				
<47	2558	450	1	Reference	638	136	1	Reference	333	47	1	Reference	203	22	1	Reference	336	61	1	Reference
47–49	2393	449	1.09	(0.94, 1.26)	637	128	0.98	(0.74, 1.29)	321	60	1.48	(0.96, 2.27)	180	39	2.05	(1.14, 3.70)	291	72	1.50	(1.01, 2.24)
50–52	4571	870	1.11	(0.98, 1.26)	1162	241	0.98	(0.77, 1.24)	644	131	1.59	(1.09, 2.32)	324	76	2.21	(1.31, 3.75)	611	102	1.04	(0.72, 1.49)
>52	3051	633	1.12	(0.97, 1.28)	840	164	0.95	(0.73, 1.24)	415	94	1.70	(1.13, 2.55)	202	48	2.36	(1.33, 4.21)	395	88	1.38	(0.94, 2.01)
Never (premenopausal)	1171	351	2.34	(1.93, 2.85)	208	47	1.17	(0.76, 1.80)	173	32	1.50	(0.86, 2.61)	100	18	1.76	(0.81, 3.80)	130	29	1.55	(0.88, 2.72)
*p*-trend^a^			0.12				0.72				0.01				0.004				0.38	
*p*-heterogeneity							0.70				0.09				0.08				0.28	

^a^The category *Never* was not included in the analysis of *p*-trend. *p for trend and OR mutually adjusted for age (50–54, 55–59, 60–64, 65–70 years at screening), body mass index (BMI) (≤22, 23–25, 26–28, >28 at screening), education (no education/primary school, high school, bachelor or master’s degree), age at menarche (9–12, 13, 14, 15–18 years), number of pregnancies (0, 1, 2, 3, ≥4), menopausal status (premenopausal, perimenopausal, postmenopausal). ^#^ *p* for heterogeneity across subtypes was determined in a case − case model (see “Methods”). *ER* estrogen receptor, *PR* progesterone receptor, *HER2* human epidermal growth factor receptor 2

Later age at first birth was associated with an increased risk of breast cancer overall and with several breast cancer subtypes (Table 2). Compared to those with an early first birth (≤20 years), women with a later age at first birth (>30 years) were at slightly increased risk of luminal A-like (OR = 1.19, 95% CI 0.99–1.43) and luminal B-like HER2-negative breast cancer (OR = 1.60, 95% CI 1.10–2.32, *p*-trend = 0.07 and 0.01, respectively). The ORs associated with the oldest age at first birth (>30 years) were also similarly elevated, but not significantly so, for HER2-positive (OR = 1.67, 95% CI 0.89–3.12) and triple-negative (OR = 1.47, 95% CI 0.88–2.47) subtypes (*p*-trend = 0.49 and 0.16, respectively). However, the test for heterogeneity comparing each subtype to luminal A-like breast cancer was not statistically significant for age at first birth.

Duration of breastfeeding was not statistically significantly associated with breast cancer overall (*p*-trend = 0.14) (Table 1) or with any of the subtypes (Table 2). Postmenopausal status was associated with a statistically significantly reduced risk of breast cancer overall (Table 1) and of luminal A-like breast cancer compared to being premenopausal (OR = 0.51, 95% CI 0.43–0.60), while no association was observed for any of the other subtypes, and all tests for heterogeneity were statistically significant. Age at menopause, comparing women >52 to women <47 years old was positively associated with risk of luminal B-like HER2-positive (OR = 1.70, 95% CI 1.13–2.55, *p*-trend = 0.01) and HER2-positive (OR = 2.36, 95% CI 1.33–4.21, *p*-trend = 0.004) subtypes (Table 2).

Duration of oral contraceptive use and use of an IUD, were associated with a slight increased risk of breast cancer overall (Table 1), with 10% elevated ORs among women with the longest duration of use. The effect estimates for long duration of oral contraceptive use were similar, but not statistically significant, for luminal A-like (OR = 1.11, 95% CI 0.96–1.29) and luminal B-like HER2-negative (OR = 1.12, 95% CI 0.81–1.54) subtypes. Women who had used an IUD for more than 10 years had an increased risk of luminal A-like breast cancer compared to never users (OR = 1.35, 95% CI 1.14–1.61) (Table 3). We observed no evidence of any clear associations for the other subtypes, and in fact a reduced OR for the luminal B-like HER2-positive (OR = 0.51, 95% CI 0.27–0.96) subtype (*p*-heterogeneity with luminal A-like breast cancer = 0.009) (Table 3).

**Table 3:** Adjusted odds ratios (OR) and 95% confidence intervals (CI) for association between breast cancer subtypes and oral contraceptives and hormone therapy

	Luminal A-like				Luminal B-like HER2-negative				Luminal B-like HER2-positive				HER2-positive				Triple-negative			
	ER+ PR+ HER2-				ER+ PR- HER2-				ER+ PR+/PR- HER2+				ER- PR- HER2+				ER- PR- HER2-			
	Controls (*n*)	Cases (*n*)	OR*	95% CI	Controls (*n*)	Cases (*n*)	OR	95% CI	Controls (*n*)	Cases (*n*)	OR	95% CI	Controls (*n*)	Cases (*n*)	OR	95% CI	Controls (*n*)	Cases (*n*)	OR	95% CI
Age at start of oral contraceptives (years)																				
14–18	1476	285	1	Reference	334	65	1	Reference	187	45	1	Reference	128	17	1	Reference	180	55	1	Reference
19–20	1462	387	1.33	(1.11, 1.59)	367	87	1.20	(0.82, 1.75)	230	49	0.90	(0.55, 1.45)	133	27	1.52	(0.75, 3.10)	206	46	0.67	(0.42, 1.07)
21–24	1959	405	1.05	(0.87, 1.25)	458	92	1.04	(0.71, 1.53)	295	45	0.62	(0.37, 1.02)	130	24	1.45	(0.69, 3.04)	253	41	0.44	(0.27, 0.74)
25–50	1993	397	0.99	(0.83, 1.20)	536	102	0.97	(0.66, 1.43)	296	58	0.80	(0.49, 1.32)	114	27	2.23	(1.06, 4.71)	258	58	0.63	(0.39, 1.03)
Never used	6715	1344	1.01	(0.86, 1.19)	1758	363	0.99	(0.71, 1.38)	876	180	0.82	(0.53, 1.27)	501	115	2.05	(1.08, 3.89)	860	165	0.54	(0.36, 0.81)
*p*-trend^a^			0.09				0.43				0.71				0.02				0.03	
*p*-heterogeneity^#^							0.60				0.52				0.07				0.02	
Duration of oral contraceptives (years)																				
Never used	6715	1344	1	Reference	1758	363	1	Reference	876	180	1	Reference	501	115	1	Reference	860	165	1	Reference
<2	1839	364	0.97	(0.85, 1.11)	482	80	0.82	(0.62, 1.08)	275	34	0.64	(0.42, 0.97)	151	18	0.47	(0.27, 0.81)	263	43	0.85	(0.58, 1.26)
2–5	1753	361	1.03	(0.90, 1.18)	435	88	1.01	(0.77, 1.33)	233	48	0.94	(0.64, 1.37)	113	17	0.61	(0.34, 1.09)	228	56	1.32	(0.92, 1.89)
6–10	1568	332	1.06	(0.92, 1.23)	388	80	1.03	(0.77, 1.37)	240	56	1.23	(0.85, 1.76)	121	35	1.07	(0.66, 1.73)	203	47	1.20	(0.82, 1.76)
>10	1266	298	1.11	(0.96, 1.29)	271	62	1.12	(0.81, 1.54)	204	45	0.95	(0.64, 1.42)	82	19	1.02	(0.56, 1.86)	149	38	1.25	(0.82, 1.90)
*p*-trend*			0.13				0.51				0.57				0.91				0.11	
*p*-heterogeneity^#^							0.61				0.10				0.003				0.56	
Age at start of intrauterine device (years)																				
14–28	612	125	1	Reference	152	28	1	Reference	82	14	1	Reference	55	12	1	Reference	88	19	1	Reference
29–35	681	143	0.97	(0.73, 1.28)	152	39	1.31	(0.75, 2.30)	93	10	0.76	(0.30, 1.90)	46	7	0.74	(0.24, 2.25)	78	25	1.42	(0.70, 2.88)
36–42	532	147	1.33	(1.00, 1.77)	114	21	0.83	(0.43, 1.58)	89	19	1.34	(0.60, 3.01)	43	7	0.75	(0.26, 2.16)	78	12	0.70	(0.31, 1.58)
43 − 50	456	121	1.16	(0.86, 1.56)	136	25	0.86	(0.46, 1.59)	70	11	1.06	(0.42, 2.70)	39	7	0.94	(0.32, 2.78)	70	14	0.94	(0.42, 2.12)
Never used	10000	2025	0.93	(0.75, 1.15)	2600	529	0.94	(0.61, 1.45)	1368	284	1.36	(0.72, 2.56)	729	153	1.10	(0.56, 2.16)	1279	267	0.94	(0.55, 1.61)
*p*-trend^a^			0.07				0.34				0.10				0.40				0.45	
*p*-heterogeneity^#^							0.22				0.12				0.20				0.38	
Duration of intrauterine device (years)																				
Never used	10000	2025	1	Reference	2600	529	1	Reference	1368	284	1	Reference	729	153	1	Reference	1279	267	1	Reference
<2	341	64	0.91	(0.69, 1.21)	83	16	0.86	(0.49, 1.52)	56	13	1.23	(0.62, 2.43)	33	5	0.84	(0.31, 2.32)	37	7	0.90	(0.38, 2.13)
2–5	432	104	1.17	(0.93, 1.48)	112	21	0.95	(0.58, 1.56)	62	11	0.96	(0.47, 1.94)	36	7	0.81	(0.34, 1.91)	57	21	1.79	(1.01, 3.16)
6–10	615	140	1.16	(0.95, 1.42)	153	40	1.37	(0.94, 2.01)	89	15	0.86	(0.46, 1.59)	53	8	0.62	(0.27, 1.43)	99	20	1.23	(0.72, 2.11)
>10	820	212	1.35	(1.14, 1.61)	176	31	0.96	(0.63, 1.46)	111	12	0.51	(0.27, 0.96)	63	12	0.73	(0.37, 1.47)	120	20	0.75	(0.44, 1.28)
*p*-trend			<0.0001				0.52				0.06				0.18				0.98	
*p*-heterogeneity^#^							0.08				0.01				0.65				0.22	
Hormone therapy use																				
Never	7411	1356	1	Reference	1762	356	1	Reference	1010	195	1	Reference	573	113	1	Reference	991	196	1	Reference
Past	4809	1086	1.27	(1.15, 1.40)	1361	276	0.98	(0.81, 1.18)	688	142	1.07	(0.81, 1.42)	319	65	0.88	(0.60, 1.30)	575	126	1.04	(0.79, 1.38)
Estrogen current	655	131	1.06	(0.86, 1.31)	162	31	0.81	(0.53, 1.23)	85	19	1.14	(0.65, 1.99)	51	12	1.12	(0.54, 2.35)	93	16	0.79	(0.43, 1.46)
Estrogen and progesterone current	345	184	2.92	(2.36, 3.62)	89	33	1.74	(1.10, 2.74)	48	18	1.67	(0.89, 3.14)	32	6	0.88	(0.33, 2.30)	49	10	0.92	(0.43, 1.98)
*p*-trend			<0.0001				0.34				0.15				0.77				0.72	
*p*-heterogeneity^#^							0.05				0.34				0.03				0.01	
Duration of hormone therapy (years)																				
Never	7411	1356	1	Reference	1762	356	1	Reference	1010	195	1	Reference	573	113	1	Reference	991	196	1	Reference
< =3	1625	306	1.09	(0.94, 1.26)	473	96	0.99	(0.76, 1.30)	237	43	0.90	(0.61, 1.34)	134	20	0.66	(0.38, 1.16)	234	41	0.77	(0.52, 1.15)
4–8	1130	240	1.21	(1.03, 1.43)	289	64	1.06	(0.77, 1.46)	155	35	1.19	(0.76, 1.87)	76	19	1.18	(0.65, 2.13)	119	25	0.99	(0.59, 1.67)
>8	1756	555	1.84	(1.61, 2.11)	485	112	1.24	(0.95, 1.61)	253	63	1.34	(0.91, 1.98)	108	23	0.93	(0.52, 1.65)	222	47	1.04	(0.69, 1.56)
*p*-trend			<0.0001				0.15				0.14				0.94				1.00	
*p*-heterogeneity^#^							0.001				0.48				0.11				0.02	
Duration of estrogen and progesterone therapy (years)																				
Never	7411	1356	1	Reference	1762	356	1	Reference	1010	195	1	Reference	573	113	1	Reference	991	196	1	Reference
< =5	1908	412	1.25	(1.09, 1.43)	545	114	1.02	(0.79, 1.32)	276	45	0.79	(0.53, 1.17)	153	30	0.92	(0.56, 1.53)	230	54	1.19	(0.81, 1.74)
> 5	1796	561	1.80	(1.58, 2.06)	496	116	1.18	(0.90, 1.54)	256	71	1.47	(1.00, 2.16)	105	29	1.04	(0.62, 1.74)	211	52	1.34	(0.89, 2.02)
*p*-trend			<0.0001				0.31				0.27				0.98				0.13	
*p*-heterogeneity^#^							0.004				0.19				0.19				0.02	

^a^The category *Never* was not included in the analysis of *p*-trend. *p for trend and OR mutually adjusted for age (50–54, 55–59, 60–64, 65–70 years at screening), body mass index (BMI) (≤22, 23–25, 26–28, >28 at screening), education (no education/primary school, high school, bachelor or master’s degree), age at menarche (9–12, 13, 14, 15–18 years), number of pregnancies (0, 1, 2, 3, ≥4), menopausal status (premenopausal, perimenopausal, postmenopausal). ^#^ *p* for heterogeneity across subtypes was determined in a case − case model (see “Methods”). *ER* estrogen receptor, *PR* progesterone receptor, *HER2* human epidermal growth factor receptor 2

Compared to women who had never used EPT, current use of EPT was associated with an increased risk (OR = 2.32, 95% CI 1.97–2.72) of breast cancer overall (Table 1) and an increased risk of luminal A-like breast cancer (OR = 2.92, 95% CI 2.36–3.62) (Table 3). The ORs for both luminal B-like subtypes were about 1.7, but this was only statistically significant for the luminal B-like HER2-negative subtype. There was no increased risk of triple-negative breast cancer with the use of EPT (*p*-heterogeneity = 0.006). Previous studies have suggested that the effect of menopausal hormone therapy may be modified by BMI, with stronger risk estimates in lean than in obese women. We therefore examined the effect of duration of EPT in different BMI strata. For breast cancer overall, the ORs associated with longer duration of EPT was significantly higher in lean (BMI <25) than in obese (BMI ≥25) women (*p* for interaction = 0.001) (Table 4). We observed similar effect modifications by BMI in luminal A-like and luminal B-like HER2-negative breast cancer. There was no evidence that BMI modified the associations between duration of EPT and risk of any of the other subtypes (Table 4).

**Table 4:** Adjusted odds ratios (OR) and 95% confidence intervals (CI) for association between breast cancer overall and subtypes of breast cancer, and the duration of estrogen and progestin therapy (EPT) by body mass index (BMI)

OVERALL breast cancer								
	BMI <25				BMI ≥25			
	Controls (*n*)	Cases (*n*)	OR*	95% CI	Controls (*n*)	Cases (*n*)	OR	95% CI
Duration of EPT (years)								
Never used	6154	1056	1	Reference	6880	1402	1	Reference
< =5	1740	316	1.20	(1.00, 1.44)	1812	420	1.13	(0.97, 1.32)
>5	1623	495	1.96	(1.64, 2.34)	1615	456	1.31	(1.12, 1.54)
*p*-trend*			<0.0001				<0.0001	
*p* for interaction			0.001					
Luminal A-like breast cancer								
Duration of EPT (years)								
Never used	3424	550	1	Reference	3892	789	1	Reference
< =5	959	176	1.36	(1.06, 1.74)	987	243	1.22	(0.99, 1.50)
>5	896	293	2.29	(1.80, 2.91)	906	270	1.38	(1.12, 1.71)
*p*-trend			<0.0001				0.002	
*p* for interaction			0.001					
Luminal B-like HER2-negative breast cancer								
Duration of EPT (years)								
Never used	850	173	1	Reference	887	178	1	Reference
< =5	257	57	1.13	(0.74, 1.71)	295	59	0.83	(0.54, 1.29)
>5	243	71	1.22	(0.79, 1.88)	256	47	0.83	(0.51, 1.35)
*p*-trend			0.34				0.37	
*p* for interaction			0.03					
Luminal B-like HER2-positive breast cancer								
Duration of EPT (years)								
Never used	450	81	1	Reference	544	113	1	Reference
< =5	137	16	0.85	(0.40, 1.81)	144	29	0.82	(0.45, 1.49)
>5	127	28	1.56	(0.67, 3.60)	130	43	1.56	(0.87, 2.78)
*p*-trend			0.44				0.24	
*p* for interaction			0.52					
HER2-positive breast cancer								
Duration of EPT (years)								
Never used	269	54	1	Reference	296	56	1	Reference
< =5	80	16	1.14	(0.44, 2.95)	77	14	1.38	(0.62, 3.08)
>5	49	13	1.53	(0.57, 4.05)	57	17	1.28	(0.56, 2.93)
*p*-trend			0.41				0.43	
*p* for interaction			0.89					
Triple-negative breast cancer								
Duration of EPT (years)								
Never used	457	94	1	Reference	522	101	1	Reference
< =5	123	20	0.96	(0.45, 2.04)	110	34	1.40	(0.79, 2.47)
>5	113	25	1.27	(0.56, 2.86)	100	27	1.34	(0.71, 2.54)
*p*-trend			0.63				0.25	
*p* for interaction			0.09					

*p for trend and ORs mutually adjusted for age (50–54, 55–59, 60–64, 65–70 years at screening), BMI (≤22, 23–25, 26–28, >28 at screening), age at menarche (9–12, 13, 14, 15–18), education (no education/primary school, high school, bachelor’s or master’s degree), number of pregnancies (0, 1, 2, 3, ≥4), menopausal status (premenopausal, perimenopausal, postmenopausal). *HER2* human epidermal growth factor receptor 2

When we added grade to the luminal A-like and both luminal B-like definitions, the results changed slightly, but were largely the same (Appendix). The largest difference was a slightly stronger effect of current EPT on the luminal A-like subtype (OR = 3.03 when grade was included in the definition versus OR = 2.90 when it was not).

Among the patients (cases), 38% (*n* = 1813) were diagnosed within a month of completing the questionnaire. Because these women could have been symptomatic when they completed their questionnaire, we ran a sensitivity analysis excluding these women. However, this did not affect the results (results not shown).

## Discussion

In this population-based study within a national screening program, we had information on 4748 patients with breast cancer, which makes it one of the largest single studies of breast cancer subtypes. The number of previous pregnancies was associated with a decreased risk, and late age at first birth was associated with an increased risk of luminal-like subtypes. Although not statistically significant, number of pregnancies and age at first birth were also associated with HER2-positive and triple-negative breast cancer. There were larger differences between subtypes with the use of exogenous hormones. Duration of oral contraceptive use and IUDs were weakly associated with luminal A-like breast cancer, while current EPT was associated with an almost threefold increased risk of luminal A-like breast cancer, but was not associated with either HER2-positive or triple-negative cancer.

There have been different methods of classifying breast cancer subtypes. In our study we used the classification from the St. Gallen meeting in 2013 [46] where we included only ER+ PR+ HER2- as luminal A, while ER+ PR- HER2- was classified as one of the luminal B definitions, while the second luminal B subtype was the one that is more commonly referred to as luminal B, i.e. ER+ and/or PR+ HER2+. In contrast, in the systematic review of 38 studies [47] they used a wider definition of luminal A (ER+ and/or PR+ and HER2-), and only one luminal B subtype (ER+ and/or PR+ HER2+). Some studies have also added on Ki67 to the definition of luminal B [40, 48, 49], which may give a more precise definition of luminal B, although Ki67 is notoriously difficult to assess [55]. Although our luminal A results were much in line with previous studies, there were some slight differences between our study and previous studies of luminal B, as discussed below.

In the current study we also examined triple-negative breast cancer (ER- PR- HER2-). Some studies have added additional markers, to better define the subset that is basal-like, by HER1+ and/or cytokeratin 5/6+ [9, 39, 40, 49, 56]. As discussed subsequently, this may explain some discrepancies between the results of different studies.

### Reproductive factors

Our observation of an inverse association between parity and luminal A-like breast cancer is consistent with the vast majority of studies as summarized in the recent systematic review of 38 studies [47] and the more recent studies that were not included in the review [7, 8, 10, 48–51]. The results for luminal B breast cancer have been less clear, with studies finding a protective or increased effect, or no effect of parity [47–50]. In the current study, parity was associated with a decreased risk of both luminal B-like subtypes. This is consistent with four out of six studies included in the systematic review [47] and three of the more recent studies [48–50]. Two of these recent studies had information on Ki-67 [48, 49]. When we added grade to the luminal A-like and luminal B-like subtypes, the parity results remained largely the same. These results suggest that there is a protective effect against luminal B, and the effect seems apparent regardless of the markers used to define the luminal B subtype.

Although not statistically significant, we found that parity was associated with a decreased risk of HER2-positive breast cancer. This is consistent with the findings in the Nurses’ Health Study [49], but inconsistent with a case–control study from the Breast Cancer Family Registry [50] and a Korean cohort study [48], which reported that parity was associated with an increased risk of the HER2-positive subtype. One of these latter studies included very few women with HER2-positive breast cancer and both studies included younger women than the current study.

However, although not statistically significant, we found that parity was associated with a decreased risk of triple-negative breast cancer.

This is inconsistent with several other studies in which parity was associated with no risk [48], or was associated with increased risk of triple-negative breast cancer [7, 47, 50]. One of the studies was in African American women who are less likely to breastfeed compared to Caucasian women [7]. Several studies have used CK5/6 and epithelial growth factor receptor (EGFR) in addition to ER, PR and HER2neu to define a basal-like subtype [9, 39, 40, 49, 56], and this may be one reason for the inconsistency between our study and these other studies. We included 386 women with triple-negative breast cancer in our study, but few of these had never been pregnant (*n* = 34) or had four or more children (*n* = 36). More notably, our study consisted of older women (aged 50–69 years). Although we did not have information on time since the last pregnancy, we can only speculate that few women in our study had had a recent pregnancy, a factor associated with increased risk of triple-negative cancer [57]. Perhaps this explains why we did not find an increased risk of triple-negative cancer with multi-parity.

We found that age at first birth (26 years or older) was associated with an increased risk of luminal-like breast cancer. This is consistent with a systematic review and [47] and two recent studies [8, 49]. However, age at first birth was only statistically significantly associated with luminal A cancer, whereas in the current study age at first birth was significantly associated with luminal A-like and luminal B-like HER2-negative subtypes. One reason for this difference might be that our definition of luminal B-like HER2-negative cancer was rather similar to the definition of the luminal A-like subtype used in the other studies. In the current study, a non-statistically significant positive association was observed between late age at first birth (31 years or older), and both HER2-positive and triple-negative breast cancer. This is consistent with the Nurses’ Health Study [49], and inconsistent with the case-control study from Korea [8]. In the latter study, the majority of women were premenopausal, whereas the current study included mainly postmenopausal women.

We found no protective effect of breastfeeding on breast cancer occurrence overall or for any subtype. This is inconsistent with the systematic review that reported that breastfeeding is associated with decreased risk of luminal A-like, luminal B-like and triple-negative subtypes [47].This may be because our study only included women above 50 years of age, and is consistent with the suggestion that the protective effect of breastfeeding is relatively time-limited, and may be seen predominantly in younger women [58–60]. Several of the more recent studies found a significant protective effect against basal-like breast cancer [7, 49–51]. These studies included both triple-negative breast cancer as in our study and basal-like breast cancer with more markers (CK 5/6 and EGFR) [49].

### Oral contraceptives, intrauterine devices, and menopausal hormone therapy use

For long duration of oral contraceptive use (>10 years), we observed a slight increase in risk of all the subtypes except for the luminal B-like HER2-positive and HER2-positive subtypes. Our positive association between duration of oral contraceptive use and triple-negative breast cancer is consistent with the systematic review [47]. However, two of the three studies in the review reported a decreased risk between oral contraceptive use and the luminal A subtype [9, 34]. These latter studies were smaller and included younger women (20–74 and <56 years old) than the current study. There are few data on IUD use. Two studies reported that IUDs were not associated with an increased risk of breast cancer [61, 62], whereas other studies report that IUDs were associated with an increased breast cancer risk [63, 64]. We observed a significant positive trend for association between duration of IUD use and breast cancer overall and luminal A-like breast cancer. However, the increased risk of luminal A-like breast cancer was only statistically significant in women using IUDs for more than 10 years. This is consistent with two Finnish studies of levonorgestrel-releasing IUDs and breast cancer [63, 64], which reported that levonorgestrel-releasing IUDs were associated with an increased risk of breast cancer. One hypothesis is that levonorgestrel-releasing IUDs have substantial progestogenic and androgenic effects [63, 64], which could contribute to this increased risk with IUD use.

We observed a large increased risk of luminal A-like and a moderate increased risk of luminal B-like breast cancer with use of menopausal hormone therapy, albeit only significantly so for the luminal A-like subtype. This is consistent with a systematic review [47] and the cohort study from the Nurses’ Health Study that investigated only one luminal B subtype, and used Ki-67 to differentiate between luminal A-like and luminal B-like subtypes [49]. There was some evidence that menopausal hormone therapy was associated with a slightly decreased risk of triple-negative breast cancer when we compared current menopausal hormone therapy users to never users. This is consistent with one of the studies in the systematic review, which was a case-control study from Washington state [35]. The latter study used the same definition of triple-negative breast cancer as the current study. Inconsistent with our result, studies from the Women’s Health Initiative [65] and the Nurses’ Health Study [40, 49] reported an association between menopausal hormone therapy and increased risk of triple-negative breast cancer. In these latter studies, they used more biomarkers (CK 5/6 and EGFR) to define the basal-like subtype and included younger women than the current study. When we looked at the duration of EPT, compared to never users, women who had used EPT for more than 5 years were at an increased risk of HER2-positive and triple-negative breast cancer. This is consistent with the analysis from the Nurses’ Health Study [49].

We hypothesized that EPT would have a stronger effect on thin women than on heavier women, and our results suggested modification of the effect of the duration of EPT when we analyzed leaner women (BMI <25) and heavier women (BMI >25) with the luminal-like subtypes of breast cancer. This is consistent with a previous population-based case–control study of women aged 55–72 years [66].

### Mechanisms and suggested subtype differences

Pregnancies have been reported to ultimately reduce plasma estrogen (estrone, estradiol and estriol) [67] and follicular-phase progesterone [68], and increase sex hormone-binding globulin [67]. Our findings suggest that both parity and combined menopausal hormone therapy may be predominantly associated with luminal-like breast tumors, with the association being strongest for the luminal A-like subtype. The effect of EPT was stronger when we added grade to the definition of luminal A cancer. We observed little effect of breastfeeding overall, and no clear subtype differences. It is possible that the effect of breastfeeding is non-hormonal, and includes changes in immune responses and apoptosis [69, 70].

Our results, together with those from other large studies, further suggest that the associations between these hormonal-related factors (parity, age at first birth, oral contraceptive use and menopausal hormone therapy use) and risk of the luminal B-like subtypes are similar to the associations between these factors and risk of the luminal A-like subtype. However, although the association with luminal A was the strongest, it is clear that EPT also increases the risk of breast cancer with bad prognosis [71].

We observed some intriguing associations with HER2-positive breast cancer. The effect of pregnancies and age at first birth were similar for the HER2-positive subtype and luminal cancer. Also, age at menopause was strongly associated with increased risk only of the HER2-positive subtype. Further, we observed a positive trend of association between age at menopause and the luminal B-like HER2-positive subtype. These results might imply that there are hormonal mechanisms involved in the expression of the HER2 protein. On the other hand, exogenous hormone use (EPT) was not associated with this subtype, suggesting that perhaps only some hormonal mechanisms play a role in HER2-positive subtypes. The contrasting results between the luminal-like subtypes and triple-negative breast cancer are also consistent with previous literature. Hormonal factors have a stronger effect on ER+ PR+ tumors, which suggests that the etiology of triple-negative cancer is different from that of the luminal subtypes. Specifically, this suggests that triple-negative tumors may not be as easily prevented hormonally.

In summary, the strongest discrepancy across subtypes was for the use of combined hormone therapy, where the effect was clearly much stronger for luminal A-like than for other cancers. Otherwise, the associations with hormonal risk factors were stronger for luminal A and B-like subtypes than for HER2-positive and triple-negative subtypes. Our results suggest that reproductive factors may to some extent be associated with HER2-positive tumors, but that triple-negative tumors have a different etiology.

### Strengths and limitations

Strengths of this study include its population-based design, the large size, being the largest single study of breast cancer subtypes conducted so far, and the availability of prospectively collected detailed information on many risk factors for breast cancer.

Although the study is the largest to date, there was still limited power for analysis of the rare breast cancer subtypes. Another weakness was the lack of molecular expression data. This may have obscured differences between the subtypes. Another possible weakness was that a subset of women were diagnosed within a month of completing the questionnaire (*n* = 1813), and could have been symptomatic when they completed their questionnaire. However, exclusion of women who were diagnosed within a month of completing the questionnaire did not affect the results (results not shown).

Women who attend screening might be more health-conscious and have a healthier lifestyle than women who do not attend. This could have contributed to obliteration of protective effects of “healthy” habits, such as an effect of breastfeeding overall. At the same time, women who attend screening are more likely to have breast cancer detected. Thus, the picture becomes complicated with these potential biases, and it is not clear how this would explain the results of this paper. The associations between well-established risk factors and overall risk of breast cancer were largely as expected. Furthermore, it is unlikely that any such bias would have differentially affected the results for different subtypes.

## Conclusions

Reproductive factors and menopausal hormone therapy use were more strongly associated with luminal-like breast cancer, but reproductive factors were also associated with HER2-positive and triple-negative breast cancer. The differences between subtypes were greatest for menopausal hormone use. Our results add to the literature showing that there are etiologic differences between luminal breast cancer subtypes and basal-like or triple-negative breast cancer subtypes, but suggest that the differences may be limited.

## Appendix

**Table 5:** Adjusted odds ratios (OR) and 95% confidence intervals (CI) for association between breast cancer subtypes and reproductive and hormonal factors with grade included in the luminal-like subtypes

Characteristics	Luminal A-like: ER+ PR+ HER2-, low and medium grade				Luminal B-like, HER2-negative: ER+ PR- HER2-, high grade				Luminal B-like, HER2 positive: ER+ PR+/PR- HER2+, low/medium/high grade				HER2-positive: ER- PR- HER2+				Triple-negative: ER- PR- HER2-			
	Controls (*n*)	Cases (*n*)	OR*	95% CI	Controls (*n*)	Cases (*n*)	OR	95% CI	Controls (*n*)	Cases (*n*)	OR	95%CI	Controls (*n*)	Cases (*n*)	OR	95% CI	Controls (*n*)	Cases (*n*)	OR	95% CI
Age at first birth (years)																				
13–20	3951	609	1	Reference	1001	42	1	Reference	553	91	1	Reference	279	54	1	Reference	495	95	1	Reference
21–22	2277	376	1.06	(0.91, 1.23)	620	32	1.31	(0.77, 2.25)	305	47	0.94	(0.62, 1.42)	170	32	0.90	(0.53, 1.53)	326	65	1.13	(0.78, 1.62)
23–25	3152	481	0.99	(0.86, 1.14)	759	32	1.42	(0.80, 2.52)	437	87	1.21	(0.85, 1.73)	222	36	0.72	(0.43, 1.20)	393	86	1.28	(0.89, 1.85)
26–30	2473	427	1.12	(0.96, 1.30)	647	25	0.71	(0.38, 1.31)	343	59	1.16	(0.78, 1.74)	216	41	0.94	(0.56, 1.55)	318	63	1.13	(0.76, 1.67)
31–50	937	196	1.27	(1.03, 1.57)	223	15	1.28	(0.58, 2.81)	127	32	1.53	(0.90, 2.60)	63	23	1.65	(0.86, 3.17)	120	29	1.36	(0.80, 2.33)
*p*-trend*			0.06				0.82				0.12				0.53				0.28	
*p*-heterogeneity^#^							0.73				0.24				0.71				0.72	
Number of pregnancies lasting 6+ months																				
0	1199	245	1	Reference	303	15	1	Reference	164	43	1	Reference	84	27	1	Reference	144	34	1	Reference
1	1596	325	0.95	(0.78, 1.15)	426	18	0.90	(0.42, 1.94)	229	41	0.58	(0.35, 0.96)	128	20	0.50	(0.26, 0.96)	224	41	0.70	(0.42, 1.16)
2	6266	1057	0.80	(0.68, 0.94)	1602	75	1.03	(0.55, 1.94)	842	178	0.78	(0.52, 1.16)	426	97	0.78	(0.47, 1.29)	813	158	0.77	(0.50, 1.18)
3	3883	572	0.67	(0.56, 0.79)	950	42	0.93	(0.47, 1.82)	528	81	0.56	(0.36, 0.86)	319	60	0.61	(0.36, 1.03)	477	117	0.98	(0.63, 1.53)
>4	1518	199	0.62	(0.50, 0.77)	383	13	0.82	(0.36, 1.87)	225	28	0.43	(0.25, 0.73)	98	19	0.60	(0.31, 1.19)	196	36	0.70	(0.41, 1.21)
*p*-trend			<0.0001				0.66				0.002				0.19				0.88	
*p*-heterogeneity							0.85				0.41				0.31				0.04	
Parous women only																				
Duration breastfeeding (months)																				
No breastfeeding	997	165	1	Reference	260	10	1	Reference	146	31	1	Reference	78	10	1	Reference	135	24	1	Reference
0–6	3145	500	0.93	(0.76, 1.15)	831	37	0.95	(0.41, 2.18)	449	71	0.67	(0.40, 1.10)	243	37	1.42	(0.63, 3.18)	467	80	1.01	(0.60, 1.71)
7–12	3108	527	1.07	(0.87, 1.31)	763	38	0.94	(0.41, 2.19)	427	91	0.84	(0.51, 1.37)	215	52	2.25	(1.02, 4.99)	359	95	1.65	(0.98, 2.78)
13–20	2583	402	0.99	(0.80, 1.24)	654	32	0.91	(0.38, 2.18)	343	75	0.94	(0.56, 1.58)	156	48	2.51	(1.11, 5.68)	308	69	1.29	(0.74, 2.23)
21–30	1700	263	1.02	(0.80, 1.30)	399	13	0.46	(0.17, 1.21)	240	37	0.67	(0.36, 1.24)	147	28	1.78	(0.74, 4.28)	222	44	1.18	(0.65, 2.15)
>30	966	154	1.11	(0.83, 1.47)	250	9	0.81	(0.26, 2.53)	121	12	0.57	(0.25, 1.29)	70	13	1.82	(0.67, 4.97)	123	24	1.19	(0.58, 2.44)
*p*-trend			0.33				0.17				0.69				0.12				0.52	
*p*-heterogeneity							0.72				0.16				0.60				0.85	
Menopausal status																				
Premenopausal	1171	291	1	Reference	208	10	1	Reference	173	30	1	Reference	100	18	1	Reference	130	29	1	Reference
Perimenopausal	1055	175	0.59	(0.47, 0.74)	225	11	0.91	(0.34, 2.42)	152	25	0.82	(0.44, 1.50)	79	20	1.41	(0.67, 2.94)	143	37	1.13	(0.64, 1.99)
Postmenopausal	12000	1932	0.50	(0.42, 0.60)	3231	142	1.06	(0.47, 2.39)	1663	316	1.05	(0.64, 1.72)	876	185	1.21	(0.66, 2.23)	1581	320	0.82	(0.50, 1.33)
*p*-trend			<0.0001				0.77				0.59				0.71				0.21	
*p*-heterogeneity							0.02				0.01				0.001				0.002	
Age of menopause (years)																				
<47	2569	367	1	Reference	640	24	1	Reference	334	44	1	Reference	204	22	1	Reference	336	61	1	Reference
47–49	2404	373	1.11	(0.94, 1.31)	639	32	1.21	(0.65, 2.27)	326	55	1.34	(0.85, 2.10)	181	39	2.06	(1.14, 3.73)	292	72	1.47	(0.98, 2.20)
50–52	4698	704	1.11	(0.96, 1.29)	1190	51	1.33	(0.76, 2.31)	663	123	1.57	(1.06, 2.33)	338	77	2.32	(1.36, 3.94)	623	102	0.99	(0.69, 1.44)
>52	3255	491	1.05	(0.90, 1.23)	871	35	1.29	(0.71, 2.34)	442	90	1.71	(1.12, 2.61)	225	48	2.38	(1.33, 4.26)	420	88	1.37	(0.94, 2.01)
*p*-trend			0.60				0.37				0.01				0.004				0.42	
*p*-heterogeneity							0.84				0.03				0.06				0.26	
Age at start of oral contraceptives (years)																				
Never used	6715	1070	1	Reference	1758	78	1	Reference	876	166	1	Reference	501	115	1	Reference	860	165	1	Reference
14–18	1476	226	0.94	(0.79, 1.12)	334	12	0.72	(0.34, 1.50)	187	44	1.24	(0.80, 1.92)	128	17	0.49	(0.26, 0.93)	180	55	1.86	(1.23, 2.81)
19–20	1462	319	1.33	(1.14, 1.55)	367	23	1.35	(0.76, 2.39)	230	47	1.09	(0.72, 1.63)	133	27	0.74	(0.45, 1.23)	206	46	1.24	(0.83, 1.85)
21–24	1959	323	1.02	(0.88, 1.18)	458	23	1.14	(0.65, 2.00)	295	43	0.78	(0.53, 1.15)	130	24	0.71	(0.42, 1.18)	253	41	0.82	(0.55, 1.22)
25–50	1993	325	1.01	(0.87, 1.16)	536	18	0.71	(0.40, 1.25)	296	54	1.01	(0.70, 1.44)	114	27	1.09	(0.66, 1.79)	258	58	1.17	(0.82, 1.67)
*p*-trend			0.41				0.63				0.56				0.70				0.91	
*p*-heterogeneity							0.77				0.54				0.08				0.02	
Duration of oral contraceptives (years)																				
Never used	6715	1070	1	Reference	1758	78	1	Reference	876	166	1	Reference	501	115	1	Reference	860	165	1	Reference
<2	1839	294	0.97	(0.84, 1.13)	482	19	0.75	(0.42, 1.34)	275	33	0.67	(0.44, 1.03)	151	18	0.47	(0.27, 0.81)	263	43	0.85	(0.58, 1.26)
2–5 years	1753	295	1.05	(0.91, 1.23)	435	15	0.73	(0.39, 1.38)	233	46	0.95	(0.64, 1.41)	113	17	0.61	(0.34, 1.09)	228	56	1.32	(0.92, 1.89)
5–10 years	1568	268	1.06	(0.90, 1.24)	388	20	1.02	(0.56, 1.85)	240	54	1.27	(0.87, 1.85)	121	35	1.07	(0.66, 1.73)	203	47	1.20	(0.82, 1.76)
>10 years	1266	232	1.09	(0.92, 1.29)	271	14	1.00	(0.49, 2.03)	204	41	0.95	(0.62, 1.44)	82	19	1.02	(0.56, 1.86)	149	38	1.25	(0.82, 1.90)
*p*-trend			0.22				0.91				0.54				0.91				0.11	
*p*-heterogeneity							0.76				0.14				0.003				0.66	
Age at start of intrauterine device (years)																				
Never users	10000	1609	1	Reference	2600	113	1	Reference	1368	266	1	Reference	729	153	1	Reference	1279	267	1	Reference
14–28	612	105	1.12	(0.89, 1.41)	152	5	1.14	(0.40, 3.24)	82	14	0.76	(0.40, 1.45)	55	12	0.91	(0.46, 1.80)	88	19	1.07	(0.62, 1.83)
29–35	681	113	0.99	(0.79, 1.23)	152	7	1.11	(0.45, 2.72)	93	10	0.62	(0.31, 1.24)	46	7	0.68	(0.27, 1.68)	78	25	1.51	(0.90, 2.55)
36–42	532	121	1.57	(1.25, 1.98)	114	5	0.61	(0.22, 1.72)	89	18	0.95	(0.54, 1.69)	43	7	0.69	(0.29, 1.61)	78	12	0.75	(0.39, 1.44)
43–50	456	96	1.32	(1.02, 1.69)	136	5	0.97	(0.33, 2.86)	70	8	0.57	(0.25, 1.28)	39	7	0.85	(0.35, 2.09)	70	14	1.01	(0.53, 1.90)
*p*-trend			0				0.66				0.13				0.31				0.89	
*p*-heterogeneity							0.79				0.07				0.21				0.40	
Duration of intrauterine device (years)																				
Never used	10000	1609	1	Reference	2600	113	1	Reference	1368	266	1	Reference	729	153	1	Reference	1279	267	1	Reference
<2	341	53	0.96	(0.70, 1.32)	83	2	0.81	(0.16, 4.02)	56	12	1.17	(0.58, 2.39)	33	5	0.84	(0.31, 2.32)	37	7	0.90	(0.38, 2.13)
2–5 years	432	89	1.27	(0.98, 1.63)	112	6	1.79	(0.62, 5.18)	62	10	0.85	(0.41, 1.76)	36	7	0.81	(0.34, 1.91)	57	21	1.79	(1.01, 3.16)
5–10 years	615	110	1.19	(0.94, 1.49)	153	4	0.49	(0.16, 1.43)	89	13	0.80	(0.41, 1.54)	53	8	0.62	(0.27, 1.43)	99	20	1.23	(0.72, 2.11)
>10 years	820	173	1.36	(1.12, 1.64)	176	10	1.41	(0.62, 3.18)	111	12	0.53	(0.28, 1.02)	63	12	0.73	(0.37, 1.47)	120	20	0.75	(0.44, 1.28)
*p*-trend			<0.0001				0.90				0.06				0.18				0.98	
*p*- heterogeneity							0.71				0.01				0.58				0.31	
Hormone therapy use																				
Never	7411	1085	1	Reference	1762	80	1	Reference	1010	183	1	Reference	573	113	1	Reference	991	196	1	Reference
Past	4809	880	1.28	(1.15, 1.43)	1361	63	1.06	(0.71, 1.59)	688	132	1.09	(0.82, 1.45)	319	65	0.88	(0.60, 1.30)	575	126	1.04	(0.79, 1.38)
Estrogen current	655	107	1.09	(0.86, 1.37)	162	5	0.77	(0.27, 2.15)	85	19	1.28	(0.73, 2.26)	51	12	1.12	(0.54, 2.35)	93	16	0.79	(0.43, 1.46)
Estrogen and progesterone current	345	150	3.03	(2.39, 3.83)	89	3	0.80	(0.21, 3.04)	48	15	1.42	(0.72, 2.78)	32	6	0.88	(0.33, 2.30)	49	10	0.92	(0.43, 1.98)
*p*-trend			<0.0001				0.80				0.19				0.77				0.72	
*p*-heterogeneity							0.02				0.17				0.04				0.005	
Duration of hormone therapy (years)																				
Never used	7411	1085	1	Reference	1762	80	1	Reference	1010	183	1	Reference	573	113	1	Reference	991	196	1	Reference
< =3	1625	246	1.10	(0.93, 1.29)	473	21	1.09	(0.62, 1.91)	237	43	1.01	(0.67, 1.51)	134	20	0.66	(0.38, 1.16)	234	41	0.77	(0.52, 1.15)
4–8	1130	197	1.19	(0.99, 1.43)	289	17	1.27	(0.66, 2.45)	155	31	1.14	(0.71, 1.83)	76	19	1.18	(0.65, 2.13)	119	25	0.99	(0.59, 1.67)
>8	1756	445	1.85	(1.59, 2.15)	485	23	1.01	(0.56, 1.80)	253	58	1.35	(0.91, 2.02)	108	23	0.93	(0.52, 1.65)	222	47	1.04	(0.69, 1.56)
*p*-trend			<0.0001				0.75				0.15				0.94				1.00	
*p*-heterogeneity							0.11				0.50				0.04				0.01	
Duration of estrogen and progesterone therapy (years)																				
Never	7411	1085	1	Reference	1762	80	1	Reference	1010	183	1	Reference	573	113	1	Reference	991	196	1	Reference
< =5	1908	321	1.22	(1.04, 1.42)	545	25	1.08	(0.62, 1.87)	276	44	0.85	(0.57, 1.27)	153	30	0.92	(0.56, 1.53)	230	54	1.19	(0.81, 1.74)
>5	1796	454	1.82	(1.57, 2.10)	496	16	0.77	(0.39, 1.50)	256	64	1.41	(0.95, 2.10)	105	29	1.04	(0.62, 1.74)	211	52	1.34	(0.89, 2.02)
*p*-trend			<0.0001				0.65				0.30				0.98				0.13	
*p*-heterogeneity							0.002				0.33				0.18				0.02	

**p* for trend and OR mutually adjusted for age (50–54, 55–59, 60–64, 65–70 years at screening), body mass index (BMI) (≤22, 23–25, 26–28, >28 at screening), education (no education/primary school, high school, bachelor’s or master’s degree), age at menarche (9–12, 13, 14, 15–18 years), number of pregnancies (0, 1, 2, 3, ≥4), menopausal status (premenopausal, perimenopausal, postmenopausal). ^#^ *p* for heterogeneity across subtypes was determined in a case–case model (see “Methods”)

## Abbreviations

BMI: body mass index
CI: confidence interval
CISH: chromogenic in situ hybridization
CRN: The Cancer Registry of Norway
EPT: estrogen and progesterone therapy
ER: estrogen receptor
FISH: fluorescence in situ hybridization
HER2: human epidermal growth factor receptor 2
IHC: immunohistochemical analysis
PR: progesterone receptor
SISH: silver in situ hybridization
